# Occupational Exposure to Urban Air Pollution and Allergic Diseases

**DOI:** 10.3390/ijerph121012977

**Published:** 2015-10-16

**Authors:** Luigi Vimercati, Maria Franca Gatti, Antonio Baldassarre, Eustachio Nettis, Nicola Favia, Marco Palma, Gabriella Lucia Maria Martina, Elisabetta Di Leo, Marina Musti

**Affiliations:** 1 Interdisciplinary Department of Medicine, Occupational Medicine “B. Ramazzini”, University of Bari Medical School, Bari 70124, Italy; E-Mails: mariafranca.gatti@gmail.com (M.F.G.); antonio.baldassarre@uniba.it (A.B.); docfavia@virgilio.it (N.F.); marcopalma3@gmail.com (M.P.); gabriellalm.martina@gmail.com (G.L.M.M.); marina.musti@uniba.it (M.M.); 2 Department of Internal Medicine and Infectious Diseases, University of Bari Medical School, Bari 70124, Italy; E-Mails: ambulatorio.allergologia@uniba.it (E.N.); elisabettadl@alice.it (E.D.L.)

**Keywords:** urban pollution, allergic sensitization, health surveillance

## Abstract

Exposure to air pollution is associated with increased morbidity from cardiovascular diseases, lung cancer, respiratory and allergic diseases. The aim of this study was to investigate allergic diseases in 111 traffic wardens compared to a control group of 101 administrative employees. All participating subjects underwent a physical examination, in which a complete medical history was taken and a dedicated allergological questionnaire administered. Spirometry, Specific IgE dosage (RAST) and skin prick tests (SPT) were done. Diagnostic investigations such as the nasal cytology, a specific nasal provocation test and rhinomanometry were also performed. Statistical analyses were performed using STATA version 11. The percentage of subjects with a diagnosis of allergy was higher in the exposed workers than in the controls. As regards the clinical tests, the positivity was higher for the group of exposed subjects. Among the exposed workers, those who worked on foot or motorcycle had a higher positivity in clinical trials compared to the traffic wardens who used the car. Our study showed a higher percentage of allergic subjects in the group of workers exposed to outdoor pollutants than in the controls. These results suggest that allergological tests should be included in the health surveillance protocols for workers exposed to outdoor pollutants.

## 1. Introduction

Air pollution is one of the main risk factors for public health in urban areas. Traffic-related pollution is one of the principal sources of particulate matter (PM), nitrous oxide, carbon dioxide, ozone and carbon monoxide [1,2], but among biological pollutants that play a particular role in the genesis of allergic disorders we must include allergizing substances such as pollens and molds.

Some studies have also investigated the role of climatic factors (e.g., barometric pressure, temperature and humidity) in triggering and/or exacerbating respiratory allergic symptoms in predisposed subjects, but the results are still poorly understood [3].

The association between air pollution and health effects seems to have been amply demonstrated [4], in terms of mortality and increased hospitalization for non allergic cardio-circulatory and respiratory diseases, whereas the effects in allergic diseases still seem to be conflicting [5], because of different results obtained until now by authors, due to the small number of studies available in literature.

The greatest evidence regards the adverse effects on the health of allergic subjects, presenting particularly in the form of asthma attacks, increased hospitalizations and drug use related to brief periods of exposure to particulate matter (PM_10_ and PM_2.5_) [6].

Studies conducted to identify a causal relationship between urban pollution and an exacerbation of asthmatic bronchial reactivity in asthma patients have yielded contrasting results: some Authors reported an increased disease burden [7,8,9], with exacerbation of the asthma attacks on days when the levels of ozone and other pollutants were higher [10] while others found no increased prevalence rate of asthma [11,12].

Oxidative stress is included among the pathogenic mechanisms invoked to explain the relation between air pollutants, many of which include oxidant components, and the onset or exacerbation of asthma. An experimental study made of air pollutants, including diesel oil exhaust fumes, found a 10% decrease in the proteasome pathway, the intracellular protein degradation system in the white blood cells, for 2 h following exposure to the aerosol. It was therefore hypothesized that oxidative stress could be the mechanism through which traffic-related air pollution exerts its effects on the intracellular regulation of the inflammatory process [13].

Several studies have also investigated a causal relationship between working activities involving exposure to outdoor pollutants and allergic diseases. Among these, Dragonieri *et al*. [14] found that the policemen exposed to traffic related air pollution presented airway neutrophilic inflammation detected by induced sputum.

Other studies investigated the role of diesel exhaust fumes in the pathogenesis of bronchial asthma [15], and demonstrated that they can induce acute inflammation of the bronchial epithelium and the recruitment of inflammatory cells, an increased expression of adhesion molecules in the vascular endothelium, as well as cytokines, mitogen-activated protein kinases and transcription factors. It has been suggested that the epithelial damage induced by the exposure to diesel exhaust fumes could cause a reduced mucociliary clearance and hence easier access of allergens to the mucosa immune system cells [16,17].

Apart from diesel particulate matter, the NO_2_ emitted by traffic can also enhance an allergic response in allergic subjects [18]. The aim of the present study was to verify a possible individual sensitization to airborne allergens in workers with occupational exposure to environmental pollutants. Traffic policemen in the city of Bari (Southern Italy), with high-density road traffic, were recruited to the study.

## 2. Materials and Methods

The research was carried out on a working population of 540 Municipal Police employees (340 traffic policemen and 200 controls). Both exposed and control subjects were selected randomly to reduce the possibility of bias in self-selection. For the inclusion into the study, a questionnaire with the physiological anamnesis and working history, including also the confounding factors, was collected for all workers, in presence of a physician.

The response rate was higher for cases (91%) than for controls (85%).

All recruited subjects, both exposed and non exposed, had a negative clinical history for previous diseases of the respiratory tract (inclusion criteria) and, only for non-exposed subjects we adopted the exclusion criteria if previously and/or currently occupationally exposed to outdoor pollutants.

The traffic policemen were assigned to the control of the flow of vehicles on roads and areas with high and medium traffic intensity and to monitor and to regulate traffic at road junctions, parking areas and traffic limited-areas. They carried out outdoor for at least 80% of their working time (7 h/day for at least 5 days a week). They were not provided with personal equipment for the protection against dust and fumes in the urban workplace. Controls were employees of the municipal police who carried out administrative and bureaucratic tasks, matched by age, gender and seniority, with non-occupational exposure to urban pollutants. In order to avoid the influence of the main confounding factors, the following subjects were excluded form the study: 2 traffic policemen and 9 controls because they had the job for less than 1 year, 10 traffic policemen and 5 controls because of a history of previous diseases of respiratory tract.

After giving informed consent to take part, all participating subjects underwent a physical examination, in which a complete medical history was taken and a dedicated allergological questionnaire administered, taking into account other allergy questionnaires, that explored family history, symptoms, past allergy problems and previous allergy evaluation and therapy. The history and questionnaire outcome was not used to classify subjects as patients [19].

All subjects agreed to the processing of their personal data and understood that this information was categorized as “sensitive data”. All subjects were informed that data from the research protocol would be treated in an anonymous and collective way, with scientific methods and for scientific purposes in accordance with the principles of the Helsinki Declaration.

After this procedure, the final sample consisted of 212 subjects, 111 exposed and 101 controls.

All the participants to the study live in Bari City and aged between 25 and 55.

Subsequently, spirometry was performed with the COSMED Pony FX (version 1.7) to assess the respiratory function and to identify any obstructive and/or restrictive deficits.

All respiratory function tests were performed by the same operator, in sitting position with the nose closed by a clip, following the American Thoracic Society (ATS) guidelines. Spirometric airflow limitation was defined as Tiffenau Index (FEV1/FVC) < 70 and/or a FEV1 < 80 of predicted values.

Specific IgE dosage and Skin Prick Test (SPT)s were performed in all subjects, both exposed and not exposed. SPTs is a method used to diagnose IgE-mediated allergic diseases.

SPTs were performed with standard panel of inhalant: Dermatophagoides pteronyssinus and farinae, cat and dog dandruff, grass pollen, olive pollen, Cupressus sempervirens, Cupressus arizonica, Parietaria judaica, Artemisia vulgaris, Platanus acerifolia, Quercus ilex, Aspergillus mix and Alternaria tenuis (Stallergenès, Milan, Italy). Histamine hydrochloride (10 mg/mL) as a positive control and physiological saline as a negative control were used. A drop of allergenic extract was applied on a surface of the forearm, than the upper layers of skin were pricked by a sterile mono-use lancet crossing the drop.

SPT reaction was considered positive if the wheal diameter was ≥ 3 mm than that provoked by negative control [20].

In addition, the specific serum IgE by ImmunoCAP System method versus inhalant allergens (*Dermatophagoides pteronyssinus* and *farinae*, *Lolium perenne*, olive pollen, *Cupressus sempervirens*, *Parietaria judaica* and *Artemisia vulgaris*) were dosed. Values higher than 0.10 (KUA/L) of specific IgE, as reported by the manufacturer (UniCAP, Thermofisher, Milan, Italy), indicate sensitization to allergen.

A diagnosis of allergy was made in subjects with positive results to specific serum IgE and/or SPT in presence of a positive clinical history for allergic respiratory symptoms [21].

Subjects with a negative clinical history but positive allergological tests were scheduled for phase 2 of the study. In accordance with the experimental protocol, they underwent a specific NPT.

In subjects with negative results to the IgE and SPT tests but a positive history for allergic symptoms, nasal cytology (using sample obtained by scraping from the medial surface of the nasal inferior turbinates using a plastic curette: Rhinoprobe; Apotex Scientific, Arlington, TX, USA) and Nasal Provocation Test (NPT) with common inhalant allergens (Lofarma, Milan, Italy) were performed.

We used rhinomanometry as method of evaluating nasal response to NPT.

If the clinical and allergological history and tests were all negative, the clinical workup and relative protocol were stopped.

A descriptive analysis of the population sample was made to calculate the mean, median, standard deviation and range of the variables investigated. Non-parametric Wilcoxon-Mann-Whitney tests were performed to compare continuous variables and chi-square Pearson tests for categorical data. A *p*-value ≤ 0.05 was considered statistically significant.

The RR was calculated by the regression test of Poisson (confidence range 95%); we used the hypersensitiveness and respiratory symptoms as variables for health outcome and smoke habit, the means of locomotion used (on foot, by motorcycle and by car), the patrolled area (center, periphery, suburbs), seniority, working hours and distance for exposure outcome.

Statistical analyses were performed using STATA version 11 Software (STATA corporation).

Our study is in accordance with good clinical practice guidelines.

Both spirometric and allergological exams were performed in agreement with the health surveillance protocol and health promotion’s campaigns (Italian Legislative Decree N° 81 of 2008, in transposition of several European Directives).

## 3. Results

In the group of exposed workers there were 76 males (68%) and 35 females (32%), and mean age was 44 years. The control group consisted of 66 males (65%) and 35 females (35%), with a mean age of 41 years. As regards smoking habit, the traffic police sample included 86 non-smokers, 21 smokers and 4 former smokers, while in the control group there were 78 non-smokers, 20 smokers and 3 former smokers. Characteristics of both groups are shown in Table 1.

**Table 1:** Characteristics of both exposed and non-exposed groups.

Characteristics		Non-Exposed	Exposed
Age			
	average	41.28	43.67
	standard deviation	10.74	41.28
	range	28–64	25–65
Smoking Habit			
	ex	3	4
	no	78	86
	yes	20	21
	**total**	**101**	**111**
Sex			
	M	66	76
	F	35	35
	**total**	**101**	**111**

Then, analyzing their work tasks, it was found that 44 subjects in the exposed group (39.6%) worked on foot, 38 (34.2%) in cars, and 29 (26.1%) on motorcycles. The work hours amounted to 6 h/day for 46.85% of the exposed subjects, and 38.7% traveled more than 40 Km/day, by car or motorcycle. In terms of job duration, 51 subjects had been working for more than 10 years, 35 between 5 and 10 years, and 25 less than 5 years.

In the exposed workers group, a diagnosis of allergy was made for 35 road traffic police (31.5%), who had a positive clinical history (symptoms) and one or more positive tests results. A diagnosis of allergy was made in 16 non-exposed subjects (15.8%) (groups I). There was therefore a statistically significant difference between Exposed and Non-Exposed (RR = 1.99 CI 95% 1.1–3.6). We also performed an adjustment for confounding variables as gender, age and smoking, obtaining a RR = 2.01 (1.07–3.78).

The 28 traffic policemen (25.2%) and 30 non-exposed subjects (29.7%) with a negative clinical history but positive allergological tests (groups II), were scheduled for phase 2 of the study. In accordance with the experimental protocol, they underwent a specific NPT.

The 10 exposed police agents (9%) and 16 controls (15.8%) that had a positive clinical history but negative allergological tests (groups III) underwent further allergological investigations such as Nasal Cytology and Rhinomanometry.

Finally, for the 38 exposed police agents (34.2%) and 39 non exposed office workers (38.6%) a diagnosis of allergic disease was excluded because they had a negative clinical history and negative allergological tests (groups IV). Table 2 shows the groups identified.

**Table 2:** Study population stratified by groups.

	Exposed		Non-Exposed	
Group	N°	%	N°	%
Group I (history + Test +)	35	31.53%	16	15.84%
Group II (history − Test +)	28	25.23%	30	29.70%
Group III (history + Test −)	10	9.01%	16	15.84%
Group IV (history − Test −)	38	34.23%	39	38.61%
**Total**	111	100.00%	101	100.00%

Normal lung function detected by spirometry was found in exposed and non-exposed subjects. As to the clinical tests, among the exposed workers there were 63 positive subjects (56.8%). In the control group there were 46 (45%) positive subjects. Thus, the number of positive subjects was higher in the exposed group but this difference was not statistically significant (RR = 1.25 CI 95% 0.82–1.85), even when taking into account confounding variables such as gender, age class and smoking habit (RR = 0.93 CI 95% 0.73–1.18). Table 3 shows clinical-instrumental test results.

**Table 3:** Clinical-instrumental test results.

		Exposed Group		Non-Exposed Group	
		N	%	N	%
	N° of Workers	111		101	
Gender	Male	76	68.5%	66	65.3%
	Female	35	31.5%	35	34.7%
Age	Average Age	43.67		41.28	
Smoking Habit	Ex	4	3.6%	3	3.0%
	No	86	77.5%	78	77.2%
	Yes	21	18.9%	20	19.8%
Spirometric Values	Mean FEV1	91.5%		92.4%	
	Mean FVC	93.6%		94.1%	
IgE Positive	Derm. Pter	37	33.3%	33	32.7%
	Derm. Farinae	34	30.6%	30	29.7%
	Lolium Perenne	23	20.7%	18	17.8%
	Olive Pollen	22	19.8%	19	18.8%
	Cupressus Sempervirens	27	24.3%	12	11.9%
	Parietaria Judaica	19	17.1%	16	15.8%
	Cat/Dog Dandruff	13	11.7%	18	17.8%
SPT Positive	Derm Pter.	24	21.6%	32	31.7%
	Derm. Farinae	22	19.8%	28	27.7%
	Cat/Dog Dandruff	11	9.9%	18	17.8%
	Grass Pollen	13	11.7%	17	16.8%
	Olive Pollen	21	18.9%	21	20.8%
	Cupressus Sempervirens	22	19.8%	14	13.9%
	Cupressus Arizonica	32	28.8%	22	21.8%
	Parietaria Judaica	16	14.4%	18	17.8%
	Artemisia Vulgaris	9	8.1%	8	7.9%
	Platanus Acerofolia	0	0.0%	2	2.0%
	Quercus Ilex	1	0.9%	1	1.0%
	Aspergillus Mix	0	0.0%	8	7.9%
	Alternaria Tenuis	3	2.7%	2	2.0%

About 66% of the Municipal police agents worked outdoors (on foot or motorcycle), as shown in Table 4. These latter agents had a higher positivity to the clinical tests than those working by car, the difference being statistically significant (*p*-value < 0.05), whereas the zone patrolled and the daily hours of work were not correlated to the tests results.

Considering the exposed police agents on foot or motorcycles separately, there was a higher percentage of clinical positive tests than in the group that worked by car or in the offices, this difference being statistically significant (Pearson chi^2^ = 8.1934 with Pr = 0.017).

**Table 4:** Hypersensitisation cases stratified by means of locomotion.

Mean of Locomotion	Hypersensitisation				
	+	% Riga	−	% Riga	Total
on Foot	22	50.0%	22	50.0%	**44**
by Car	18	47.4%	20	52.6%	**38**
by Motorcycle	23	79.3%	6	20.7%	**29**
	**63**	**56.7%**	**48**	**43.3%**	**111**

As regards the allergological questionnaire, positive results were obtained in 51 (45.9%) of the exposed and 16 (15.8%) of the non-exposed subjects. Positivity to the allergological questionnaire was prevalent in the exposed workers group, with a statistically significant difference (RR = 2.9 CI 95% 1.65–5.08). Even when taking into account the confounding variables, there was a higher percentage of positive answers to the questionnaire in the exposed subjects group, again with a statistically significant difference (RR = 2.79 CI 95% 1.54–5.07). In both groups, the symptoms most commonly referred were conjunctivitis and allergic rhinitis.

In the questionnaire, among the exposed workers the job description and zone patrolled were not associated with the subjective answers.

The specific NPT included in the experimental protocol, which should have been performed in 28 agents, was actually performed in 22 because 6 did not comply with the second study phase, while it was performed in the 30 controls (groups II). At the end of this test, positive results were obtained in 2 traffic police agents and only one control police agent. In this phase, nasal cytology and rhinomanometry were also performed, in 10 exposed agents and 16 non exposed subjects (groups III), and yielded negative results.

## 4. Discussion

Air pollution is one of the main risk factors for public health in urban areas, due to long-term high levels of airborne fine particulate matter exposure that may affect respiratory health and impair pulmonary function.

Our study showed a higher percentage of allergic subjects in the group of workers exposed to outdoor pollutants than in the controls. Particularly, a diagnosis of allergy was made in 35 traffic policemen and 16 non-exposed subjects with a statistically significant difference between the two groups. Spirometry tests did not reveal clinically significant respiratory problems, unlike in reports by other authors, which found a significant reduction in spirometry parameters in the exposed traffic warden groups as compared to the controls [22,23,24].

The clinical tests showed a prevalence of positive results in the exposed workers (56.8%) as compared to the controls (45.5%), but this difference was not statistically significant. When considering the exposed police agents on foot or motorcycles separately, there was a higher percentage of clinical positive tests than in the group that worked by car or in the offices, this difference being statistically significant.

These results are in agreement with what our research group has previously observed [25], in a smaller sample of Municipal police agents. Also in this case, about 60% of the exposed workers were positive to at least one of the allergometric tests.

In addition, our results are in accordance with the findings of Proietti *et al.* [26], who observed a higher prevalence of respiratory symptoms and allergic sensitization in a group of traffic policemen exposed to pollutants, compared with a non-exposed group. As regards subjective symptoms, investigated by means of the questionnaire, positive results were more prevalent among the exposed workers than the controls, this difference being statistically significant. This finding seems to be in agreement with what was observed by Gao *et al*. who showed an increase of respiratory symptoms in road traffic agents, due to chronic exposure to outdoor chemical pollutants [27].

By contrast, such results were not confirmed by Obaseki *et al.* in a group of Municipal police officers in Nigeria [28].

In literature there are still few studies concerning this occupational risk that may have potential impact on occupational and public health issues related to polluted urban areas. We run a cross-sectional study to evaluate the prevalence of allergic diseases in workers occupationally exposed to urban pollution, compared to a group of worker non-occupationally exposed. Our study shows an important correlation between the occupational exposure to urban pollutants and the exacerbations and/or enhancement of allergic manifestations and highlights, also, the importance of early diagnosis to avoid severe pathological conditions. On this basis we suggest the opportunity to adopt preventive measures, such as personal protection equipment against dust and fumes in the urban workplace.

We do recognize, indeed, some limitations, such as the lack of environmental air quality data from Regional Environmental Protection Agency. Further epidemiological studies, on larger samples and environmental air quality data provided, are thus required to better understand and define the role of urban pollution, both traffic-related and biological, in inducing allergic respiratory diseases.

In conclusion, in our study we found that 60% of workers exposed to road traffic were positive to clinical allergological tests, and a diagnosis of allergic disease was made in about half of these. On this basis, we suggest that work in an outdoor environment a mid dense traffic in industrialized zones is a condition that favors the onset of allergic type manifestations. These were particularly observed in exposed subjects working on foot or motorcycle, who showed a statistically significant higher positivity to the clinical tests than the exposed subjects working by car. The possibility of making an early diagnosis of sensitization, even before an overt disease picture emerges, would allow preventive measures to be adopted in order to at least limit the later development of clinically significant allergic manifestations. In the light of our findings, we suggest that allergological tests should be included in the health surveillance protocols for workers exposed to outdoor pollutants.

## 5. Conclusions

We run a cross-sectional study to evaluate the prevalence of allergic diseases in 111 traffic wardens compared to a control group of 101 administrative employees. Our study showed a higher percentage of allergic subjects in the group of workers exposed to outdoor pollutants than in the controls. These results suggest that allergological tests should be included in the health surveillance protocols for workers exposed to outdoor pollutants. Further epidemiological studies, however, on larger samples and environmental air quality data provided, are thus required to better understand and define the role of urban pollution, both traffic-related and biological, in inducing allergic respiratory diseases.
